# Functional Polymer Solutions and Gels—Physics and Novel Applications

**DOI:** 10.3390/polym12030676

**Published:** 2020-03-18

**Authors:** Bing Du, Florian J. Stadler

**Affiliations:** College of Materials Science and Engineering, Shenzhen Key Laboratory of Polymer Science and Technology, Guangdong Research Center for Interfacial Engineering of Functional Materials, Nanshan District Key Lab for Biopolymers and Safety Evaluation, Shenzhen University, Shenzhen 518055, China; dubing@szu.edu.cn

Recent years have seen significant improvements in the understanding of functional soft matter. Advances in organic chemistry have identified a wide variety of possible interactions, ranging from hydrophobic interactions, e.g., based on cyclodextrin or hydrophobic end-capping, host–guest interactions, and multiple hydrogen bonding to metal–ligand bonding, such as for terpyridines and catechols. Those functional moieties can link polymer chains, usually in aqueous solution, and thus assemble these solutions into gels. These discoveries of the last ca. 30 years by chemists worldwide have made it possible to understand biological soft matter significantly better, as well as to create our own soft materials with tailored properties, such as a pH-controlled behavior. Their current and future applications are often focused on the biomedical field, particularly on drug release and tissue engineering. However, functional polymer solutions and gels can also be envisioned for many other applications. For example, functional nanofibers electrospun from solution can also be used for advanced applications. The resulting nanofibers combine an incredible highly specific surface area with an excellent performance as membranes with high flux, good separator selectivity, as well as extraordinary selective absorption for both functional nanoparticles and pollutants in water.

This Special Issue of *Polymers* attracted contributions from several diverse fields of polymers which can only exemplarily illustrate the broadness of the topic of polymer solutions and gels.

Polymers with special moieties, leading to thermoresponsive and stimuli-responsive behavior, was one of the main topics.

Yan et al. [1] studied the interactions between tacticity of poly (N-iso-propylacrylamide) PNIPAM and 1-butyl-3-methylimidazolium bis(trifluoromethanesulfonyl) imide ([BMIM][TFSI]) ionic liquid, in which a higher isotaxy of PNIPAM leads to a higher amount of interaction and, consequently, to more temperature stable gels, i.e., to an increased upper critical solution temperature. Interestingly, the results show that it is possible to have a decoupling of gelation and turbidity, which is counterintuitive and expands upon earlier knowledge on the physicochemical behavior of PNIPAM in ionic liquids [2]. These results show the relevance of ionic liquids for the solubilization of polymers, which has revolutionized the unwrapping of tightly packed crystalline cellulose [3,4,5,6].

Polymer solutions with catechol functionalities were shown to significantly influence thermoresponsive behavior as well as the end groups, which could be modeled statistically, demonstrating the combined effects of the end groups derived from the rather hydrophobic RAFT agents and catechol groups [7], which has led to further elucidation as well as confirmed previous observations on the properties of thermoresponsive polymers containing catechol moieties [8,9]. This work also extends on previous reports on PNIPAM-based polymer with other structures [10,11,12]. Similarly to catechols, terpyridine groups—as one species of metal–ligand complexing functionalities—are used as a terminal group of self-assembling di-block copolymers which exhibit an unusually fast self-healing behavior as well as a highly concentration and ion-type-dependent rheology [13].

Pure polymer physics was also represented in the theoretical study of Wang [14], which studied the drag reduction effect in polymer solutions, an effect which has been a focal point of interest for both experimental and theoretical polymer physicists for a long time [15,16,17], as drag reduction promises to be a technology that can contribute to the international climate crisis by reducing energy consumption for pumping fluids.

The realm of environmental technology was represented by Luo et al. [18], who introduced a way to selectively adsorb highly environmentally polluting chromium (VI) ions that are often produced during leather production [19]. This work introduces a nonwoven able to adsorb chromium but also to release it and, thus, allowing for regeneration of the adsorbent. In comparison to many adsorption approaches based on nanoparticles, nonwovens have the advantage of macroscopic size and, thus, easier handling as a filter-like material.

The biomedical applications in the Special Issue were represented by several contributions. Guan et al. [20] demonstrated the usability of alginate–acrylamide hydrogels for the production of vascular grafts—hydrogels that can be used for blood vessel repair, leading to similarly good mechanical performance as their natural porcine counterparts. Steffi et al. [21] developed a new way to improve bone-forming cell differentiation by using a strontium-doped titania tannic acid polyphenol layer on titanium for implants. Such a coating of implants could improve the interface between the implant and the bone, especially in the case of osteoporosis. Skwira et al. [22] contributed to the Special Issue in the broad field of drug delivery with their paper investigating the release properties of ciprofloxacin antibiotic from special silica-based composites with ethylcellulose and polydimethylsiloxane.

Li et al. [23] showed a biomedical-related use of hydrogels—they demonstrated how gallic acid (GA) could accumulate from red ginseng by adsorption on chitosan-based hydrogels. GA is a natural polyphenolic molecule, interesting for its antioxidant, anti-inflammatory, anticancer, and antiviral activities [24]. 

Khan et al. [25] showed one example of the use of polymer gels as dosimeters, providing a high-resolution equivalent of tissue in quality control of radiation therapy. Warman et al. [26] reported the development of a device for the tomographic imaging of radio-fluorogenic gels (RFGs), which allows for scanning the 3D distribution of radiation in a gel.

Asphalt binders are another type of gel-like material covered in this Special Issue. Yu et al. [27] showed how efficiently styrene–butadiene–styrene (SBS) tri-block copolymers are sufficiently UV-stable as an asphalt binder for permanently improving the properties of asphalt. Gong et al. [28] contributed to the same stream of research by investigating the rheological behavior of these asphalt–SBS mixtures, which complements a research line of polymer–asphalt mixtures for improving rheological properties [29,30].

Electrospinning technology is one of the most efficient and straightforward approaches to fabricate the fibrous membrane or scaffold [31]. Through the powerful tension, various solutions based on polymers or their composite are drawn and instantaneously solidified into micro-/nanoscale fibers. Owing to the nanoscale of the fibers, the electrospun products possess an extremely high ratio surface, conjunct network structure, and quite large porosity, providing plenty of possibilities to expand versatile materials. In particular, the fibrous structure can remarkably reduce the density of the materials; thus, it can effectively “soften” the substance with a lightweight feature. Many researchers develop copious types of novel soft materials, which have been widely utilized for tissue scaffold [32,33], wound dressing [34], fibrous membrane for catalyst [35], energy cell [36], and electrochemical [37] and mechanical [38] sensors. These active developments profit from the fascinating matrix based on nanofibrous structure, which extensively adapts to many fields as supporting materials. On the other hand, the electrospun membrane can also play a crucial role as one relative independent structural component because of its excellent intrinsic properties. In this Special Issue, Monteserín et al. [39] reported on interesting layer-by-layer epoxy resin composites reinforced by electrospun nanofiber veils. They included electrospun material made of polyamide 6 modified with TiO_2_ nanoparticles into carbon fiber/epoxy composite as a single structure. From this work, it was observed that the nanofibers could effectively improve the flexural stress at failure and fracture toughness of the composite. When the fibers were modified with TiO_2_, the composite exhibited new antibacterial performance, which widened the application of the material.

The field of functional polymer solutions and gels has, so far, experienced a lot of attention from chemists, who are making numerous interesting, complex systems whose physical behavior is very complex. Hence, while polymer chemistry also including functional moieties has reached a certain level of maturity, main topics remain which are lacking a good understanding of the physics of such systems. The clear application direction for functional polymer solutions and gels is towards biomedical applications, which automatically means that the questions to be answered are rather complex as, in most cases, they involve interactions of complex functional polymer materials with even more complex—especially biological—systems. Classical polymer physics on melts and solutions without functional moieties has reached maturity after being intensively researched from ca. 1950 until the present date. Functional polymers were systematically introduced to research much later, and it will certainly take several decades until the same level of understanding is reached for the physics of functional polymers. The future of physics and applications of functional polymers will be highly diverse, as is shown in this Special Issue. We expect that special emphasis will be paid on tailoring the properties of the polymers to a particular application direction, as well as to gradually increase the fraction of modified natural polymeric systems in comparison to classical synthetic polymer systems.

Functional polymer solutions and gels are often targeted for high-value applications, especially in the biomedical field. The complexities of these fields, owing to the multidimensional property profile of these materials interacting with very complex systems, also means that classical characterization methods are no longer sufficient for totally capturing the essential parameters of the system. As a consequence, we expect to see the development of combined methods, where two classical methods are merged together to yield 2 or more kinds of measurements simultaneously. Examples of these which the editors are familiar with include the combination of rheology with spectroscopic methods (nuclear magnetic resonance (NMR), Raman spectroscopy, Fourier transform infrared spectroscopy (FTIR), and dielectric spectroscopy). Furthermore, further improvement of existing methods will become necessary—one recent example being non-vacuum scanning electron microscopy (SEM), useful for investigating living biological samples or fast chip calorimetry, improving traditional speed limitations of dynamic scanning calorimetry of ca. 1–2 K/s by several orders of magnitude. Furthermore, we will see the development of completely new methods as well, which will suit needs of the scientific community. 

Lastly, as one of the primary areas of functional soft materials is the biomedical area, increasing collaborations with industrial partners will become necessary as the regulatory requirements for testing the safety of materials for in vivo use—i.e., applying the material to human or animal patients—are strongly regulated, requiring extensive and expensive tests that research institutions usually cannot pay for without strong support from industrial partners.
